# Genomic Regions From an Iranian Landrace Increase Kernel Size in Durum Wheat

**DOI:** 10.3389/fpls.2019.00448

**Published:** 2019-04-18

**Authors:** Francesca Desiderio, Leila Zarei, Stefania Licciardello, Kianoosh Cheghamirza, Ezatollah Farshadfar, Nino Virzi, Fabiola Sciacca, Paolo Bagnaresi, Raffaella Battaglia, Davide Guerra, Massimo Palumbo, Luigi Cattivelli, Elisabetta Mazzucotelli

**Affiliations:** ^1^Council for Agricultural Research and Economics, Research Centre for Genomics and Bioinformatics, Fiorenzuola d'Arda, Italy; ^2^Department of Agronomy and Plant Breeding, Razi University, Kermanshah, Iran; ^3^Council for Agricultural Research and Economics, Research Centre for Cereal and Industrial Crops, Acireale, Italy

**Keywords:** *T. durum*, landrace, QTL, kernel size, kernel weight

## Abstract

Kernel size and shape are important parameters determining the wheat profitability, being main determinants of yield and its technological quality. In this study, a segregating population of 118 recombinant inbred lines, derived from a cross between the Iranian durum landrace accession “Iran_249” and the Iranian durum cultivar “Zardak”, was used to investigate durum wheat kernel morphology factors and their relationships with kernel weight, and to map the corresponding QTLs. A high density genetic map, based on wheat 90k iSelect Infinium SNP assay, comprising 6,195 markers, was developed and used to perform the QTL analysis for kernel length and width, traits related to kernel shape and weight, and heading date, using phenotypic data from three environments. Overall, a total of 31 different QTLs and 9 QTL interactions for kernel size, and 21 different QTLs and 5 QTL interactions for kernel shape were identified. The landrace Iran_249 contributed the allele with positive effect for most of the QTLs related to kernel length and kernel weight suggesting that the landrace might have considerable potential toward enhancing the existing gene pool for grain shape and size traits and for further yield improvement in wheat. The correlation among traits and co-localization of corresponding QTLs permitted to define 11 clusters suggesting causal relationships between simplest kernel size trait, like kernel length and width, and more complex secondary trait, like kernel shape and weight related traits. Lastly, the recent release of the *T. durum* reference genome sequence allowed to define the physical interval of our QTL/clusters and to hypothesize novel candidate genes inspecting the gene content of the genomic regions associated to target traits.

## Introduction

Durum wheat (*T. turgidum* L. var. *durum*) is a major crop in Mediterranean regions with a total of about 14 million hectares cultivated worldwide. Commercial wheat cultivars have a rather narrow genetic base (Van de Wouw et al., 2010) therefore investigation and exploitation of new genetic diversity is a fundamental requirement for modern breeding programs. Landraces, the locally adapted germplasm as result of the natural and farmers' selection, represent interesting genetic materials, they usually exhibit a high genetic diversity with relevant allele variations including rare variants and/or potentially new alleles (Lopes et al., 2015).

The development of high yielding wheat cultivars is a major objective of modern breeding programs. Since grain yield is a complex trait, it is often dissected in two main components that are kernel weight, expressed as 1,000 kernel weight (TKW), and number of seeds per square meter resulting from the number of spikes per unit area and number of kernels per spike. Kernel dimensions, as kernel length (KL) and kernel width (KW), greatly influence the TKW. Moreover, especially for durum wheat, kernel size and shape also influence the test weight (TW), which, in turn, has an effect on semolina yield (Gegas et al., 2010). For these reasons, increasing TKW and TW are main targets in wheat breeding, in addition to total yield. Larger kernels not only impact on grain yield but also have favorable effects on seedling vigor and early growth (Peng et al., 2003). These traits are quantitative and complex, highly influenced by the environment (E) and display high Genotype × Environment interactions (GxE). Modern durum wheat varieties exhibit large kernels and rather uniform seed size, because of domestication and breeding for increased yield and TW. On the contrary, durum wheat landraces show a much greater variability for kernel size and shape (Moore, 2015; Liu et al., 2017).

The understanding of the genetic and molecular determinants of grain size and grain shape might provide valuable information on genetic diversity and corresponding markers to be used for improving grain yield. The most advanced genetic knowledge on the genetic factors controlling grain size and shape is available in rice where many genes have been functionally characterized. An update about genetic pathways controlling kernel size and weight in rice and Arabidopsis has been recently reported in Li and Yang (2017). Some genes (for instance: *D1, D61*, and *SRS5*) have pleiotropic effects on organ growth, including a reduction in seed size in the corresponding mutants, due to alteration of phytohormones signaling (Yamamuro et al., 2000; Ashikari et al., 2005; Segami et al., 2012). Others (for instance: *GW2, GS2, GS5, GLW7, GIF1*) appear to specifically affect grain morphology (Song et al., 2007; Wang et al., 2008; Li et al., 2011; Hu et al., 2016; Si et al., 2016). At the cellular level, increase of the grain size could be a consequence of an increase in cell number, such as for the activity of *D1* and *GS5*, or of cell size expansion, as for the role of *D61* and *GLW7*, or of both as observed for *GS2*.

The direct translation of genetic knowledge gained from rice to wheat allowed the identification of several orthologs. As in rice, *TaGW2*, encoding an E3 RING ligase (Su et al., 2011; Simmonds et al., 2016), is a negative regulator of grain size and weight (Hong et al., 2014), and showed natural allelic variation in extensive studies in both tetraploid and hexaploid wheat (Su et al., 2011; Qin et al., 2014; Jaiswal et al., 2015; Simmonds et al., 2016). Similarly, allelic variation at *TaGS-D1*, the wheat homolog of the rice *GS3* (Wang et al., 2012), and at *TaTGW6*, an enzyme related to the auxin metabolism (Hanif et al., 2015; Hu et al., 2016), showed main effects on TKW and kernel size. *TaGS5* is a positive regulator of grain size (Ma et al., 2016) and *TaCwi*, homolog of *GIF1*, encodes a cell wall invertase with effects on TKW (Jiang et al., 2015). Other genes have been found in wheat as related to kernel weight, they include *TaSAP-A1, TaGS1a, 6-SFT-A2, TaSus1*, and *TaSus2* (Jiang et al., 2011; Chang et al., 2013; Guo et al., 2013; Hou et al., 2014; Yue et al., 2015). All these genes except *TaSAP-A1* have specific roles during the grain filling.

Many studies have been conducted to identify quantitative trait loci (QTL) associated to kernel traits, TKW above all, but also parameters related to kernel size in common wheat (Sun et al., 2008; Gegas et al., 2010; Ramya et al., 2010; Tsilo et al., 2010; Prashant et al., 2012; Maphosa et al., 2014; Rasheed et al., 2014; Williams and Sorrells, 2014; Wu et al., 2015; Kumar et al., 2016; Cheng et al., 2017; Su et al., 2018; Würschum et al., 2018). In these studies, some QTLs for TKW co-localized with QTL of kernel size, thus confirming also at genetic level the positive correlation between grain size and grain weight. Furthermore, a co-location of yield related traits was also found with QTL for flowering time and plant height suggesting pleiotropic effects on fundamental agronomic traits (Gegas et al., 2010; Bogard et al., 2011). In tetraploid wheat, only two studies unravel the genetic bases of kernel size (Russo et al., 2014; Golan et al., 2015), but much more identified regions related to kernel weight (Maccaferri et al., 2016; Kidane et al., 2017; Roncallo et al., 2017; Soriano et al., 2017; Mangini et al., 2018). All findings have been collected by a global metaQTL analysis which summarized and projected all known QTLs on the durum wheat reference genome providing a tool for comparison between QTLs and candidate genes (cv Svevo; Maccaferri et al., 2019).

The current study was designed to identify novel regions of the durum wheat genome controlling kernel related traits in a RIL population derived from a cross between the Iranian cultivar Zardak and the Iranian landrace Iran_249. For this purpose, we developed a high-density genetic map, and conducted a QTL mapping whose results were physically mapped on the recently published durum wheat reference genome (Maccaferri et al., 2019). The results provide the physical position of QTLs directly on the durum wheat pseudomolecules and a list of candidate genes laying within the QTL confidential regions.

## Materials and Methods

### Genetic Materials

A population of 118 F_7−8_ RILs, derived from a cross between the landrace accession “Iran_249” originated from Western Iran, and the old cultivar “Zardak” from the Iranian Kermanshah province, was used in the current study. A leaf of each line was ground using the Retsch_MM300 Mixer Mill instrument, then the DNA was isolated and purified with the Wizard_Magnetic 96 DNA Plant System (Promega) following the manufacturer's instructions.

### Field Experiments and Phenotypic Evaluation

Seed increase was done in the experimental farm of the CREA-Research Centre for Genomics and Bioinformatics in Fiorenzuola d'Arda (Italy). The RIL population and the two parents were evaluated in Libertinia (Sicily island, southern Italy) during the 2013–2014 (L14) and 2014–2015 (L15) seasons, and in Fiorenzuola d'Arda (northern Italy) in 2014–2015 (F15), thus providing phenotypic data for three environments.

In each environment, a randomized complete block design with three replications was used; the experimental units consisted of 1.8 m^2^ in Libertinia and 3 m^2^ plot in Fiorenzuola d'Arda. Trials were fertilized following the standard agronomic practices for each location, weeds were chemically controlled. Supplementary Table 1 reports the details about field experimental conditions and relevant environmental parameters. Heading date (HD) was recorded as number of days from the April 1st to the time when 50% of tillers within a plot have spike emerged from the flag leaf sheet. Test Weight (TW) was recorded for each plot/environment. After several months of storage at constant temperature and humidity, three samples of 100 kernels were randomly chosen from the seed bulk of each plot/experiment and weighted to calculate the corresponding Thousand Kernel Weight (TKW). One 100 kernel punch for each plot/experiment was randomly sampled out and used for batch scanner imaging. Then through image analysis by the software Winseedle pro^1^ (2011 Regents Instruments Inc., Canada) kernels were measured for several descriptors of seed morphology as reported in Table 1.

**Table 1:** Kernel morphological traits evaluated through image analysis by WinSeedle software.

**Traits**	**Description**
Length (L, mm)	Line connecting the two farthest points on the projected image perimeter
Width (W, mm)	The maximum width perpendicular to length
Perimeter (P, mm)	The length of an object's projected area boundary
Area (A, mm^2^)	The two-dimensional projected area of a three-dimensional object
Curvature (C)	Defined as (a/b), where (a) is a perpendicular distance from the center of the object at the point of maximum straight width to the straight length and (b) is the straight length
WL ratio (WL)	Width to length ratio
Form coefficient (FC)	Is 4^*^A/P2 where A = cell area and P = cell perimeter. It can take values between 0 and 1, 1 being a perfect circle and 0 a filiform object (perfect line)

### Statistical Analysis of Phenotypic Data

For each environment and trait, the frequency distribution of the RIL phenotypic data was evaluated and analysis of variance (ANOVA) was performed. Overall data were analyzed by fitting a model by the REstricted Maximum Likelihood (REML) method to assess significance of Genotype (G), environment (E), and Genotype × Environment interaction (GxE). Broad sense heritability (H) was calculated according to Nyquist (1991): H = $\delta_{\text{G}}^{2}$/[$\delta_{\text{G}}^{2}$ + $\delta_{\text{GE}}^{2}$/E) + $\delta_{\text{e}}^{2}$/rE)], where $\delta_{\text{G}}^{2}$ is the genetic variance, $\delta_{\text{GE}}^{2}$ is the GxE interaction variance, $\delta_{\text{e}}^{2}$ is the residual variance, E is the number of environments, and r the number of replicates. $\delta_{\text{G}}^{2}$ was calculated as (MS_G_ – MS_GxE_)/*n* where MS_G_ is the genotype mean square and MS_GxE_ is the mean square of GxE. All these statistical analyses were conducted by using JMP version 7 software (SAS Institute Inc., 2007).

Pearson's correlation coefficients were calculated for all trait combinations based on data recorded for each year/environment, and using overall data across the three environments using the standard cor.test function in R. The significance of correlations was assessed with the *t*-test implemented in the cor.test function.

For each trait, QTL analysis was performed based on mean values of the three replicates for each single environment.

### Molecular Marker Analysis

Both simple sequence repeat (SSR) and single-nucleotide polymorphism (SNP) molecular markers were used to analyze the parental lines and the RILs.

The parental lines were screened with a total of 360 SSR markers selected from the published wheat map (Röder et al., 1998; Eujayl et al., 2002; Gupta et al., 2002; Guyomarc'h et al., 2002; Sourdille et al., 2003; Peng and Lapitan, 2005; Song et al., 2005; Xue et al., 2008). The PCR and fragment analysis were carried out as described in Desiderio et al. (2014).

Genotyping for SNPs was performed at the Trait Genetics Laboratory (Gatersleben, Germany) with the Infinium iSelect 90K wheat SNP BeadChip array (Illumina Inc., San Diego, USA), which contains 81,587 functional markers (Wang et al., 2014).

### Linkage Analysis

Linkage analysis was performed using CarthaGene software (de Givry et al., 2005) with a logarithm of odds (LOD) score threshold of 9.0, maximum distance of 20 cM and the Kosambi mapping function to calculate map distances (Kosambi, 1944). The linkage groups obtained were assigned to chromosomes by comparing markers of the generated maps to the high-density consensus durum map (Maccaferri et al., 2015). Within each linkage group, the best order of markers and the genetic distances were established using different CarthaGene functions: “build,” “greedy,” “flips,” and “polish.” All mapped markers were tested for the expected 1:1 segregation ratio using a Chi squared (χ2) goodness-of-fit test.

### QTL Analysis

QTL mapping was conducted with the R/qtl module of the R statistical computing package (Broman et al., 2003). For each trait, an initial QTL scan was performed using simple interval mapping with a 1-cM step (Lander and Botstein, 1989) and the position of the highest LOD was recorded. A genome-wide significance level of 5% was calculated after 1,000 permutations (Churchill and Doerge, 1994). The position and the effect of the QTL were then estimated using the multiple imputation method (Sen and Churchill, 2001) by executing the “sim.geno” command, followed by the “fitqtl” command. To search additional QTLs, the “addqtl” command was used. If a second QTL was detected, “fitqtl” was used to test a model containing both QTLs and their interaction effect. If both QTL remained significant, the “refineqtl” command was used to re-estimate the QTL positions based on the full model including both QTLs. QTL interactions were analyzed and the significant locus combinations are reported based on F value. The additive effects of QTLs were estimated as half the difference between the phenotypic values of the respective homozygotes.

The confidence interval (CI) of each QTL was determined as proposed by Darvasi and Soller (1997). The QTLs were named according to the rule “trait.gb + chromosome.locus number.”

### Analysis of Physical Regions Carrying QTLs Related to Kernel Traits

The most significant QTLs identified in the present study were projected on the *T. durum* reference genome sequence (cv. Svevo) (Maccaferri et al., 2019). Peak markers and flanking markers corresponding to the CIs were located on the reference genome based on the Blast matches of the corresponding SNP's nucleotide sequences. Whenever the marker was a singleton and/or found similarity hit within the unassembled fraction of the Svevo genome (chromosome 0), the marker was searched on the consensus durum map (Maccaferri et al., 2015), the cosegregant markers from the consensus were identified and the corresponding sequence localized by Blast on the Svevo genome. This approach was used to roughly locate the QTLs on the reference genome for three different comparison analyses. Firstly, the likely position of the identified QTLs was compared with that of durum wheat homologs of common wheat and rice cloned genes whose function is known to be associated to kernel related traits. Secondly, the physical position of the identified QTLs was compared with QTLs previously genetically mapped and published in tetraploid wheat for the same traits and recently anchored to the durum reference genome by Maccaferri et al. (2019). Finally, the physical region underlined by the most significant QTLs was inspected to identify candidate genes, their functional annotation, and the expression data available for the homologous genes in bread wheat.

Toward this end, durum genes were annotated via blast2GOPRO (Götz et al., 2008) using as queries proteins run against *viridiplantae* database (NCBI non-redundant protein dataset; available at FIGSHARE (https://figshare.com/s/2629b4b8166217890971).

Next, best reciprocal hit (BRH) blasts of durum wheat (cv. Svevo) CDS queries (longest representative isoforms for each gene in the physical region of interest) were conducted against a database consisting of bread wheat (Chinese Spring) CDS (longest representative isoforms for each gene; only genes located in the chromosome homologous to Svevo's query genes chromosome). The bread wheat best hits (filtered for a percent identity threshold of at least 90%) were subsequently used as queries for blasts (blast2; version 2.2.26) against the WheatExp database at https://wheat.pw.usda.gov/WheatExp/. Blasts were fine-tuned by testing several parameters (gapped vs. ungapped blasts and various score penalties for gap opening and gap extension). Finally, blast results were again filtered for a minimum identity of 90%. The expected chromosome location of hits as well as the consistency of their annotation with respect to original Svevo queries annotated with blast2GOPRO was evaluated. To be able to compare the expression profile of all the genes mapping under a specific QTL and to represent these data into a heat map, the z-scores of the FPKM log mean values have been calculated.

## Results

### Phenotypic Evaluation

The two parents, the cultivar Zardak and the landrace Iran_249, and the RILs were evaluated for traits related to kernel morphology and size (Table 1), grain weight, and for heading time in 3 years × environment combinations (Libertinia 2014 -L14- and 2015 -L15- Fiorenzuola d'Arda 2015 -F15).

**Figure 1:**
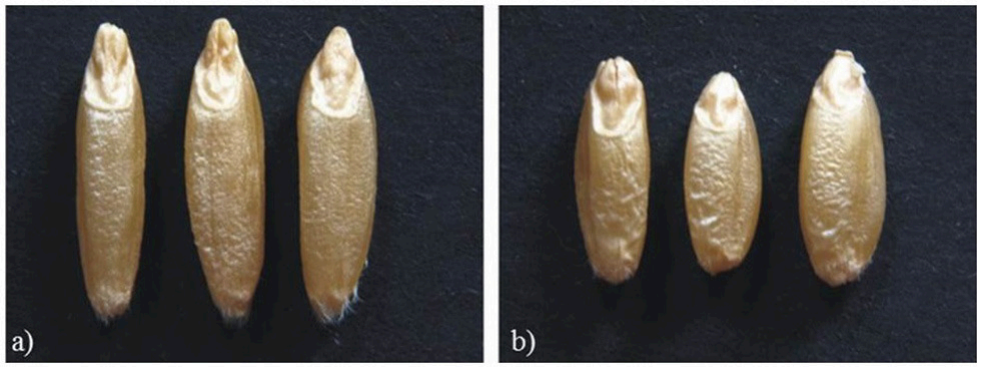
Kernel morphology of Iran_249 **(a)** and Zardak **(b)**.

**Figure 2:**
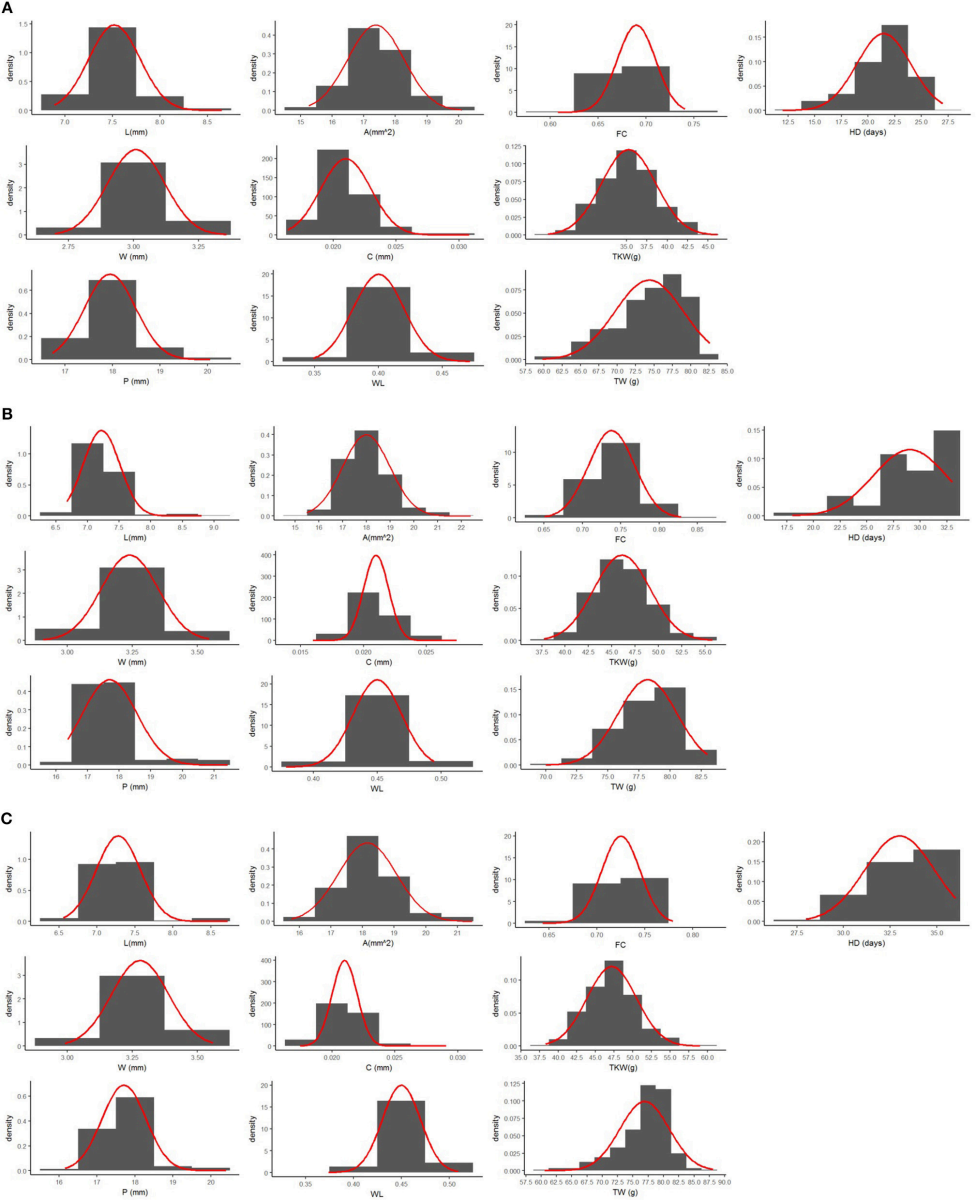
Frequency distribution for all the traits analyzed in this study. The normal distribution was represented as solid red line. Mean data for each environment **(A)** L14, **(B)** L15, and **(C)** F15 have been reported.

Mean values of Zardak, Iran_249, and RILs across the three environments are reported in Table 2, single environment data are in Supplementary Table 2. The two parents showed significant differences for kernel length, perimeter, area and shape related traits (WL, FC) in all environments, while for TKW only in L15. In detail, kernels of Iran_249 were longer and not significantly but generally narrower and heavier compared to those of Zardak *cv* (Figure 1; Table 2), while kernels of Zardak had a higher degree of roundness (FC, WL) and a higher test weight, as a consequence. Data about the RILs population showed a continuous variation and a normal distribution for most of the traits, suggesting a polygenic inheritance (Figure 2). For kernel width, transgressive segregation was observed in both directions, while for kernel length, perimeter and area only RILs with kernels shorter/smaller than the worse parent were present within the population. Consequently, RILs producing kernels with a roundness degree higher than the better parent were reported. Interestingly, some RILs showed values higher than the better parent for TKW, and TW. Overall, this evidence suggests the presence of superior QTL alleles for TKW and TW in both parents, likely supported by larger kernels and higher grain roundness.

**Table 2:** Summary of phenotypic data and variation parameters for parental lines and RILs for kernel shape (C, curvature; WL, width length ratio; FC, Form coefficient), size (L, length; W, width; P, perimeter; A, area), and weight related traits (TKW, Thousand kernel weight; TW, Test weight), and heading date (HD).

**Trait**	**Iran_249**	**Zardak**	*P*-value^*^	**RIL mean**	**RIL min**	**RIL max**	**CV%**	**MS_**G**_**	**MS_**GxE**_**	**H**
L	10.02	7.581	^*^	7.339	6.706	8.726	1.6	0.577	0.030	0.94
W	3.09	3.185	ns	3.179	2.865	3.489	2.2	0.040	0.013	0.65
P	22.74	18.169	^*^	17.790	16.420	20.631	2.2	2.349	0.358	0.82
A	23.08	18.505	^*^	17.844	15.502	21.319	3.1	4.099	0.758	0.79
C	0.022	0.021	ns	0.021	0.017	0.029	1.8	1.09 E-05	3.2 E-06	0.66
WL	0.309	0.420	^*^	0.434	0.367	0.492	1.8	2.64 E-03	1.72 E-04	0.92
FC	0.57	0.702	^*^	0.713	0.634	0.783	2	2.77 E-03	3.2 E-04	0.86
TKW	48.93	43.136	ns	42.843	33.816	53.640	5	34.36	11.77	0.62
TW	64.39	73.167	^*^	76.453	63.467	84.567	4.7	23.31	13.84	0.34
HD	29.7	28.3	ns	27.8	19.33	32	4.8	37.18	3.38	0.88

*Mean data across the three environments have been reported*.

* *Significant difference at 0.05% among parents based on t-test; CV, coefficient of variation; MS_G_, genotype mean square; MS_GxE_, mean square GxE; H, broad sense heritability*.

Variation for the phenotypic measures was assessed by ANOVA for each single environment and for the overall dataset, evaluating the effects of G, E and GxE (Table 2; Supplementary Table 3). In each single environment the variability for replications was significant for almost all traits, but much more of the variation was attributed to the genotype effect. Considering overall data across the three environments, all effects were significant for all traits. However, although the strong environment effect, the genotype variability was higher than GxE component for all traits, with the exception of the TW. As a consequence, high values of broad sense heritability were obtained for kernel size and shape related traits, ranging from 0.65 to 0.94, with generally lowest values for kernel width and curvature, and highest values for kernel length (Table 2). Moderate to low heritability values were calculated for TKW and TW (0.62 and 0.34, respectively). Finally, a high heritability (0.88) was found for heading date. Based on the highly significant GxE interaction showed by some traits, QTLs were determined using the mean values of the three replicates for each environment.

**Figure 3:**
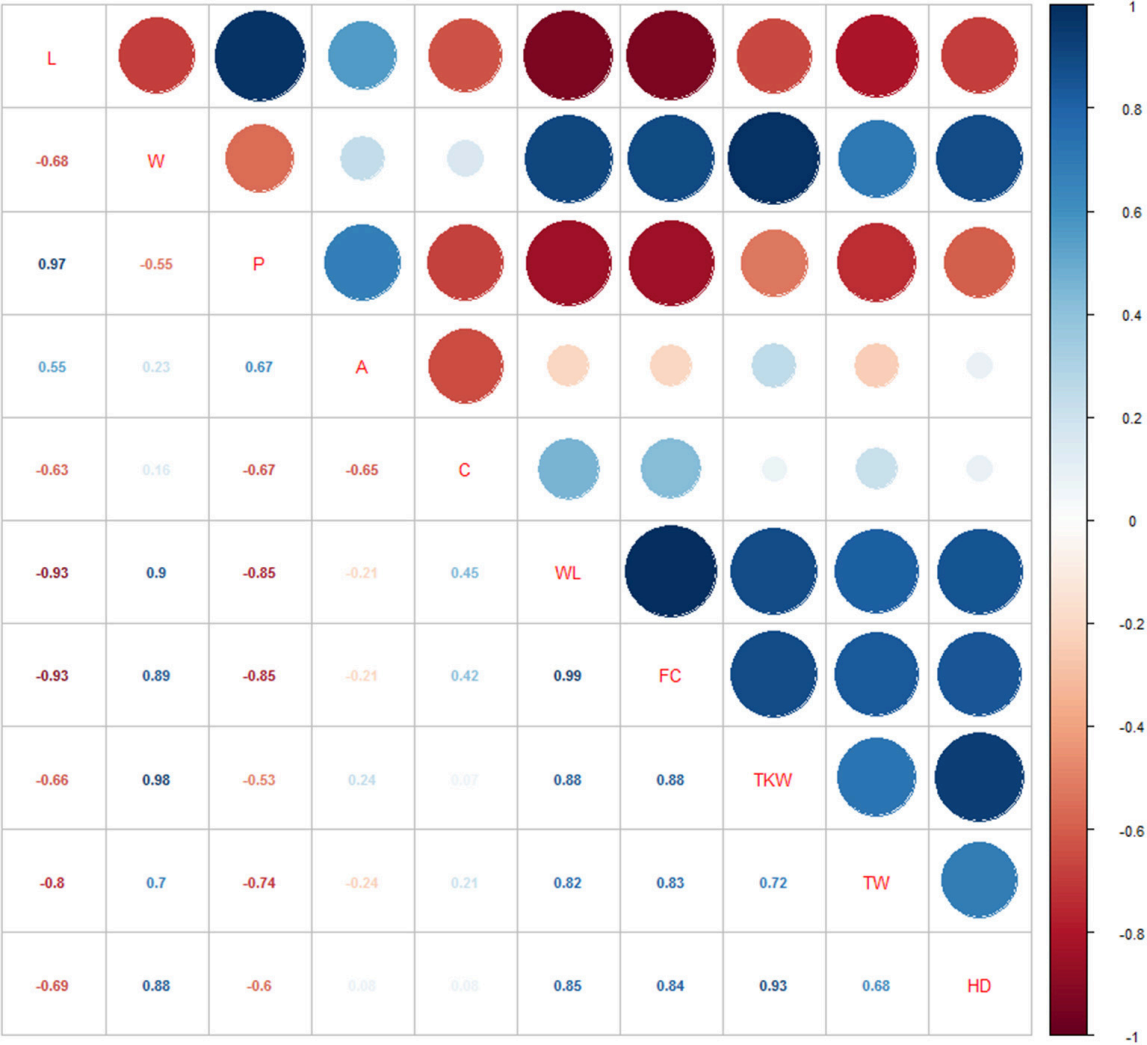
Pearson correlations among the phenotypic traits analyzed using overall data.

Pearson's correlation coefficients between all possible couple of target traits were calculated based on both single-environment data (Supplementary Table 4; Supplementary Figure 1), and overall dataset of the three environments (Supplementary Table 4; Figure 3). As expected, some traits were inherently correlated, like perimeter vs. length (*r* = 0.97), and WL vs. FC (*r* = 0.99). TKW and TW showed a high positive correlation with kernel width (*r* = 0.98 and *r* = 0.7, respectively), and kernel roundness as showed by WL (*r* = 0.88 and 0.82) and FC traits (*r* = 0.88 and *r* = 0.83), while they had negative correlations with seed length (*r* = −0.66 and −0.8, respectively). However, considering single environment data, a significant positive correlation was found between L and TKW in L15. Finally, HD was negatively correlated to L and P (*r*-values ranging from −0.6 to −0.7) and positively associated to traits about kernel width (W, WL, *r* = 0.88 and 0.85, respectively), and kernel weight related traits (TKW: *r* = 0.93, TW: *r* = 0.68).

### Molecular Analysis and Map Construction

The Zardak × Iran_249 genetic linkage map integrated both SSR and SNP markers. Out of 360 SSRs used to screen the parental lines, 87 (24%) were polymorphic between parents and were tested on the whole segregating population. Within the 81,587 markers of the 90k iSelect Infinium, 5,591 SNPs failed the hybridization and were discarded, while 8,220 (10.8%) were polymorphic between the two parents. Within the polymorphic marker set, we further removed markers showing more than 10% missing values and markers with a minor allele frequency (MAF) significantly deviating from the expected 1:1 ratio (MAF < 0.3). After these checks, 6,452 high-quality SNP markers represented the valuable SNP data set. On the overall, 6,539 polymorphic loci (comprising 87 SSR and 6,452 SNP markers) were therefore identified and used for the construction of the molecular marker map. After elimination of the unlinked loci, the genotype data relating to 6,195 informative marker loci were assembled into 18 linkage groups corresponding to the 14 durum wheat chromosomes, for a total of 977 unique loci (Table 3 and Supplementary Table 5). Two linkage groups were identified for chromosomes 1B, 2B, 6A and 6B.

**Table 3:** Distribution of molecular markers in the chromosomes and in the homeologous groups of the Zardak × Iran249 map.

**Chromosome**	**N^**°**^ of linkage group**	**Total marker**	**Map lenght (cM)**	**Marker density (cM/marker)**
1A	1	359 (76)	231.7	0.65 (3.05)
1B	2	794 (109)	246.2	0.31 (2.26)
2A	1	390 (59)	189.4	0.49 (3.21)
2B	2	529 (78)	236	0.45 (3.03)
3A	1	456 (72)	314.7	0.69 (4.37)
3B	1	471 (62)	155.7	0.33 (2.51)
4A	1	196 (41)	166.9	0.85 (4.07)
4B	1	296 (52)	165.1	0.56 (3.18)
5A	1	366 (63)	228	0.62 (3.62)
5B	1	519 (81)	229.9	0.44 (2.84)
6A	2	469 (66)	186.8	0.40 (2.83)
6B	2	563 (67)	117.4	0.21 (1.75)
7A	1	316 (73)	249.9	0.79 (3.42)
7B	1	471 (78)	166.8	0.35 (2.14)
Total	18	6,195 (977)	2884.5	0.47 (2.95)
Genome A	8	2,552 (450)	1567.4	0.61 (3.48)
Genome B	9	3,643 (527)	1317.1	0.36 (2.5)

*Within brackets, we reported the number of non-cosegregant markers and the marker density calculated accordingly*.

The overall length of the map was 2,884.5 cM with individual chromosome genetic length ranging from 314.7 cM (chromosome 3A) to 117.4 cM (chromosome 6B) and average chromosome length of 206.04 cM. The total number of mapped loci per chromosome ranged from 196 (chromosome 4A) to 794 (chromosome 1B) with an average of 442.5 loci. The genome-wide marker density was 0.47 cM, varying from 0.21 cM (chromosome 6B) to 0.85 cM (chromosome 4A).

Considering the two sub-genomes (A and B), genome B showed a higher number of loci (3,643) and a higher marker density (mean of 0.36 cM/marker), while genome A the longer map length (1,567.4 cM, Table 3).

### QTL Mapping Analysis for Kernel Size

QTL analysis was performed for traits related to kernel size, shape and weight, and HD, using phenotypic data from single environments (L14, L15 and F15). Overall, 94 QTLs distributed on all chromosomes were identified, in addition to 16 epistatic interactions (Tables 4A,B). Chromosomes 6B and 2B reported the highest QTL frequency (24 and 19, respectively). The kernel length identified the highest number of QTLs (17), followed by WL ratio (14), and perimeter (11). QTLs for the same trait, identified in different environment and with overlapping CIs or QTL peak at < 20 cM were considered the same (Maccaferri et al., 2008). Upon this merging, we identified a total of 31 different QTLs and 9 QTL interactions for kernel size (L, W) and the correlated measures (P, A), and 21 different QTLs and 5 interactions for kernel shape (C, WL, and FC; Table 4).

Table 4QTLs **(A)** and their interactions **(B)** detected in Zardak × Iran249 segregating population for traits related to kernel morphology (L, W, P, A, C, WL, FC), kernel weight (TKW, TW), and heading date (HD).

**Table:** 

**QTL name**	**Traits**	**Environment**	**Chr**.	**LG**	**cM**	**LOD**	***R*^**2**^ (%)**	**CI start (cM)**	**CI end (cM)**	**Additive effect**
**(A)**										
QA.gb-2A	A	F15	2A	1	177.6	5.29	13.52	172.50	182.70	−0.35
QA.gb-2B	A	F15	2B	1	47.3	11.78	34.44	45.30	49.30	−0.56
QA.gb-2B	A	L15	2B	1	57.5	5.40	11.10	51.30	63.70	−0.28
QA.gb-5B.1	A	L15	5B	1	85.2	8.30	18.10	81.40	89.00	−0.36
QA.gb-5B.2	A	F15	5B	1	179.5	4.11	10.27	172.80	186.20	−0.29
QA.gb-6B.1	A	L15	6B	1	0.5	4.35	15.26	0.00	4.99	−0.37
QA.gb-6B.2	A	L14	6B	1	53.8	7.63	24.15	50.96	56.64	−0.56
QA.gb-6B.2	A	F15	6B	1	54.2	4.20	14.46	49.46	58.94	−0.10
QA.gb-6B.3	A	L14	6B	2	8.6	3.94	11.58	2.69	14.51	0.17
QA.gb-6B.4	A	F15	6B	2	35.1	3.52	11.95	29.37	40.83	−0.28
QC.gb-1A	C	L15	1A	1	93.8	3.57	10.37	87.10	100.50	0.00
QC.gb-1B	C	F15	1B	1	27.9	7.35	18.88	24.20	31.60	0.00
QC.gb-2B	C	L14	2B	1	37.5	6.52	18.97	33.90	41.10	0.00
QC.gb-2B	C	F15	2B	2	30.4	4.57	11.09	24.20	36.60	0.00
QC.gb-6B	C	F15	6B	2	5.5	3.50	12.78	0.14	10.86	0.00
QC.gb-6B	C	L15	6B	2	6.0	5.81	20.30	2.63	9.37	0.00
QC.gb-6B	C	L14	6B	2	11.8	4.52	16.17	7.56	16.04	0.00
QFC.gb-1B.1	FC	F15	1B	1	2.7	7.09	14.07	0.00	7.60	0.01
QFC.gb-1B.2	FC	L14	1B	1	144.9	17.34	30.27	142.60	147.20	0.00
QFC.gb-2B	FC	F15	2B	1	61.9	6.48	12.70	56.50	67.30	0.01
QFC.gb-2B	FC	L14	2B	1	76.2	3.15	4.13	59.50	92.90	0.00
QFC.gb-6B.1	FC	L14	6B	1	0.0	5.49	19.29	0.00	3.55	0.01
QFC.gb-6B.1	FC	F15	6B	1	0.0	4.27	12.91	0.00	5.30	0.01
QFC.gb-6B.2	FC	F15	6B	2	2.0	3.37	10.00	0.00	8.85	0.01
QFC.gb-6B.3	FC	L15	6B	2	34.7	2.37	8.83	26.94	42.46	0.01
QFC.gb-7A	FC	L14	7A	1	20.1	12.23	19.31	16.52	23.68	0.07
QFC.gb-7B	FC	L15	7B	1	60.3	4.36	13.52	55.20	65.40	0.01
QFC.gb-7B	FC	F15	7B	1	77.5	9.34	20.15	74.10	80.90	0.01
QHD.gb-2A	HD	L14	2A	1	61.8	10.91	14.64	57.10	66.50	−1.23
QHD.gb-2A	HD	F15	2A	1	61.8	9.22	15.65	57.40	66.20	−0.80
QHD.gb-2B	HD	L15	2B	1	19.8	15.80	40.65	18.10	21.50	−1.80
QHD.gb-2B	HD	L14	2B	1	24.1	7.85	9.89	17.10	31.10	−0.62
QHD.gb-2B	HD	F15	2B	1	24.1	9.66	16.54	19.90	28.30	−0.83
QHD.gb-3B	HD	L15	3B	1	140.0	7.79	16.95	135.90	144.10	−1.09
QHD.gb-4B	HD	F15	4B	1	19.3	6.13	9.77	12.20	26.40	−0.57
QHD.gb-5A	HD	F15	5A	1	208.7	5.57	8.77	200.80	216.60	0.55
QHD.gb-5B	HD	L14	5B	1	86.5	10.08	13.30	81.30	91.70	−1.05
QHD.gb-5B	HD	L15	5B	1	86.5	3.67	7.34	77.10	95.90	−0.67
QHD.gb-7A	HD	L14	7A	1	20.1	8.67	11.10	13.88	26.32	−0.91
QL.gb-2B.1	L	L15	2B	1	57.5	3.60	7.14	47.80	67.20	−0.07
QL.gb-2B.2	L	L14	2B	1	78.5	6.86	8.47	70.30	86.70	−0.07
QL.gb-2B.2	L	F15	2B	1	78.5	13.45	9.40	71.20	85.80	−0.09
QL.gb-4A.1	L	L14	4A	1	23.2	7.85	9.90	16.20	30.20	−0.11
QL.gb-4A.1	L	F15	4A	1	23.2	12.64	8.68	15.20	31.20	−0.10
QL.gb-4A.2	L	L14	4A	1	154.5	9.91	13.05	149.20	159.80	0.13
QL.gb-4A.2	L	F15	4A	1	154.5	30.03	30.33	152.20	156.80	0.16
QL.gb-4B	L	L14	4B	1	110.7	7.00	8.69	102.80	118.60	−0.07
QL.gb-4B	L	F15	4B	1	110.7	15.47	11.28	104.60	116.80	−0.13
QL.gb-5B	L	L15	5B	1	205.8	5.80	12.10	200.10	211.50	0.09
QL.gb-6B.1	L	L15	6B	1	0.0	5.89	18.01	0.00	3.80	−0.13
QL.gb-6B.1	L	F15	6B	1	0.5	8.47	27.42	0.00	3.00	−0.18
QL.gb-6B.1	L	L14	6B	1	1.0	5.68	14.77	0.00	5.64	−0.11
QL.gb-6B.2	L	L14	6B	2	2.0	6.83	18.18	0.00	5.77	−0.12
QL.gb-6B.3	L	F15	6B	2	8.2	3.68	10.82	1.87	14.53	0.04
QL.gb-7B	L	L14	7B	1	63.8	13.46	19.08	60.20	67.40	−0.17
QL.gb-7B	L	F15	7B	1	63.8	20.28	16.41	59.60	68.00	−0.16
QP.gb-2A	P	F15	2A	1	32.4	9.27	20.12	29.00	35.80	−0.35
QP.gb-2B.1	P	F15	2B	1	57.5	6.33	12.93	52.20	62.80	−0.22
QP.gb-2B.1	P	L14	2B	1	61.9	4.62	9.49	54.60	69.20	−0.16
QP.gb-2B.2	P	L15	2B	1	174.2	12.92	32.30	172.10	176.30	−0.43
QP.gb-3A	P	L15	3A	1	134.2	3.45	7.10	124.50	143.90	−0.20
QP.gb-6B.1	P	L15	6B	1	0.0	6.24	21.51	0.00	3.18	−0.32
QP.gb-6B.1	P	L14	6B	1	1.0	5.41	14.68	0.00	5.67	−0.22
QP.gb-6B.1	P	F15	6B	1	1.0	4.94	17.54	0.00	4.90	−0.26
QP.gb-6B.2	P	L14	6B	2	2.0	5.84	16.00	0.00	6.28	−0.21
QP.gb-7A	P	L15	7A	1	108.7	6.86	15.13	113.26	113.26	−0.21
QP.gb-7B	P	L14	7B	1	55.9	5.78	11.92	50.10	61.70	−0.18
QTKW.gb-1B	TKW	L14	1B	1	66.5	7.63	22.46	63.40	69.60	1.08
QTKW.gb-2B	TKW	L15	2B	1	46.8	3.90	10.00	39.90	53.70	−0.87
QTKW.gb-3B	TKW	F15	3B	1	130.1	5.82	17.60	126.20	134.00	−1.32
QTKW.gb-5B	TKW	L14	5B	1	75.2	5.41	15.21	70.70	79.70	−0.86
QTKW.gb-5B	TKW	F15	5B	1	75.2	4.19	12.27	69.60	80.80	−1.00
QTKW.gb-5B	TKW	L15	5B	1	85.2	8.00	22.36	82.10	88.30	−1.26
QTW.gb-6A.1	TW	L15	6A	2	54.1	5.30	18.70	50.40	57.80	−0.75
QTW.gb-6A.2	TW	F15	6A	2	97.4	5.39	18.97	93.80	101.00	1.11
QW.gb-1B	W	L14	1B	1	4.5	9.64	27.66	2.00	7.00	0.04
QW.gb-3A	W	L15	3A	1	175.1	3.10	9.14	167.50	182.70	0.02
QW.gb-5A	W	L14	5A	1	53.6	5.75	15.22	49.10	58.10	0.03
QW.gb-5B	W	L15	5B	1	75.2	5.90	18.45	71.50	78.90	−0.04
QW.gb-6A	W	F15	6A	2	97.4	5.60	19.63	93.90	100.90	0.04
QWL.gb-1B	WL	F15	1B	1	2.7	8.17	13.24	0.00	7.90	0.01
QWL.gb-1B	WL	L14	1B	1	4.5	8.84	12.24	0.00	10.10	0.01
QWL.gb-1B	WL	L15	1B	1	4.5	9.10	17.70	0.60	8.40	0.01
QWL.gb-2B	WL	F15	2B	1	61.9	7.84	12.70	56.50	67.30	0.01
QWL.gb-2B	WL	L14	2B	1	71.3	7.90	10.73	64.90	77.70	0.01
QWL.gb-2B	WL	L15	2B	1	85.6	4.75	8.45	77.40	93.80	0.01
QWL.gb-4A	WL	L14	4A	1	23.2	6.81	9.04	15.60	30.80	0.01
QWL.gb-6A	WL	F15	6A	2	97.0	6.40	10.00	90.10	103.90	0.01
QWL.gb-6B.1	WL	L14	6B	1	0.0	3.37	10.76	0.00	6.36	0.06
QWL.gb-6B.2	WL	L15	6B	2	2.0	5.70	19.94	0.00	5.43	0.01
QWL.gb-6B.2	WL	F15	6B	2	14.9	5.65	19.79	11.44	18.36	0.01
QWL.gb-7B	WL	L14	7B	1	55.9	21.91	40.15	54.20	57.60	0.01
QWL.gb-7B	WL	L15	7B	1	55.9	11.25	22.88	52.90	58.90	0.01
QWL.gb-7B	WL	F15	7B	1	55.9	6.37	9.95	49.00	62.80	0.01

**Table:** 

**QTL interaction**	**Traits**	**Environment**	**LOD**	***R*^2^ (%)**	**Additive effect**
**(B)**					
QL.gb-4A.2*QL.gb-6B.1	L	L14	8.11	10.29	−0.12
QL.gb-4A.2*QL.gb-6B.1	L	F15	25.9	23.8	−0.18
QL.gb-4B*QL.gb-6B.1	L	L14	6.21	7.57	0.09
QL.gb-5B*QL.gb-6B.1	L	L15	5.2	10.77	−0.1
QP.gb-2B*QP.gb-7A	P	L15	4.63	9.76	0.24
QP.gb-2A*QP.gb-6B.1	P	F15	3.91	7.62	0.23
QC.gb-2B*QC.gb-6B.2	C	L14	2.62	7.04	0.00049
QC.gb-1A*QC.gb-6B.2	C	L15	2.79	7.96	0.00049
QFC.gb-1B.2*QFC.gb-6B.1	FC	L14	11.6	18.06	0.01
QFC.gb-1B.1*QFC.gb-6B.2	FC	F15	3	5.45	−0.005
QFC.gb-1B*QFC.gb-7A	FC	L14	5.43	7.45	0.0053
QHD.gb-2A*QHD.gb-7A	HD	L14	6.17	7.51	−0.82
QHD.gb-2B*QHD.gb-3B	HD	L15	3.81	7.65	−0.9
QA.gb-6B.2*QA.gb-6B.4	A	F15	3.15	10.64	0.343
QL.gb-6B.1*QL.gb-6B.3	L	F15	3.46	10.11	−0.114
QA.gb-6B.2*QA.gb-6B.3	A	L14	3.53	10.28	−0.366

#### Kernel Length

Ten QTLs were found to be significantly associated with kernel length (L, Table 4). Among them, QL.gb-6B.1 was reported in all environments, while other five QTLs were reported in the two environments L14 and F15, on chromosomes 2B, 4B, and 7B, and two QTLs on chromosome 4A (QL.gb-4A.1, QL.gb-4A.2). For all these QTLs, excepted for QL.gb-4A.2, the landrace Iran_249 contributed the allele for longer kernels. Major QTLs were QL.gb-4A.2 and QL.gb-6B.1, which showed up to 30.3 and 27.4% of phenotypic explained variance (PEV), respectively, thus defining confidence intervals narrower than 5 cM. Out of four different epistatic effects among L-related QTLs, QL.gb-6B.1 had environmentally stable relationships with QL.gb-4A.2, which explained further phenotypic variation of 8.1–25.9%.

#### Kernel Width

Five QTLs were associated with kernel width (W) on chromosomes 1B, 3A, 5A, 5B, and 6A, but none of them were conserved among environments. The region that explained the highest value for LOD and phenotypic variance (9.6 and 27.7%, respectively) was detected on chromosome 1B, QW.gb-1B (Table 4), with a confidence interval of 5cM. Zardak contributed the allele for larger kernel for all QTLs, except for the region identified on chromosome 5B based on data from L15 (QW.gb-5B, with *R*^2^ = 18.45%).

#### Kernel Perimeter

Eight QTLs were identified for kernel perimeter (P) on chromosomes 2A, 2B, 3A, 6B, 7A, and 7B. QP.gb-6B.1 was identified based on phenotypic data recorded from all environments, while QP.gb-2B.1 was reported in two environments (L14 and F15). QP.gb-6B.1 had from 14.7 to 21.5% of PEV and showed epistatic interaction with the region QP.gb-2A. QP.gb-2B.1 phenotypic variation ranged from 9.5 and 12.9% for L14 and F15 data analysis, respectively. However, the major QTL QP.gb-2B.2, with a confidence interval of 4.2 cM and around 32% PEV, was obtained based on L15 phenotypic data only and showed the highest additive effect value (0.43). In addition, this QTL showed epistatic interaction with QP.gb-7A, thus explaining a further 10% quote of PEV. For all these QTLs, the alleles for increased perimeter were contributed by Iran_249.

#### Kernel Area

Eight QTLs were detected for kernel area (A) on four different chromosomes, 2A, 2B, 5B, 6B, but only the QA.gb-6B.2 was reported in two environments (L14, F15). This QTL explained up to 24.1% of PEV and an additional quote of 10% resulted from the interaction with other two different regions identified on the same chromosome (QA.gb-6B.3 and QA.gb-6B.4). The QTL with the largest effect was identified on chromosome 2B based on F15 data. It was named QA.gb-2B.1 and explained 34.4% of phenotypic variation. The parent landrace Iran_249 contributed the positive allele for all QTLs associated to kernel area, except for QA.gb-6B.3 (Table 4).

### QTL Mapping Analysis for Kernel Shape

The analysis of three kernel shape parameters, the curvature (C), the WL ratio and the form coefficient (FC), discovered a total of 21 different QTLs and 5 QTL interactions (Table 4).

#### Curvature

Five QTLs were associated with kernel curvature (C), but only QC.gb-6B was stable across the three environments and showed the highest PEV (up to 20.3%). For all QTLs identified, with the only exception of QC.gb-2B.2 detected using data from F15, Zardak positively contributed for increased curvature, as a combination of greater width and/or shorter length.

#### WL Ratio

Seven QTLs associated with width/length phenotypic variability were found where Zardak contributed the allele with the positive effect on the target trait (Table 4). Notably, QWL.gb-1B, QWL.gb-2B, and QWL.gb-7B were stable across the three environments. In detail, QWL.gb-7B registered the highest LOD and *R*^2^ values based on data from L14 (21.91 and 40.15%, respectively), thus defining a confidence interval of 3.4 cM. QWL.gb-1B had LOD values comprised between 8.17 and 9.1 and explained a phenotypic variation ranging from 12.2 to 17.7%. About the region on chromosome 2B, the data from F15 identified the highest LOD and *R*^2^ values (LOD = 7.9, *R*^2^ = 12.7%). The region QWL.gb-6B.2 conserved across environments L15 and F15 showed till 20% of PEV.

#### Form Coefficient

Out of nine QTLs associated with FC, three were identified across two environments and located on chromosomes 2B, 6B and 7B (named as QFC.gb-2B, QFC.gb-6B.1, QFC.gb-7B, respectively). The major conserved QTLs were QFC.gb-7B and QFC.gb-6B.1, which explained around 20% PEV each. Overall, the cultivar Zardak contributed the positive allele at all loci, with the only exception of another major QTL, detected on chromosome 1B using data from L14 and explaining 30.3% of phenotypic variance (QFC.gb-1B.2). Additional quote of explained variance was retrieved by the interactions among QTLs detected, particularly for QFC.gb-1B.2 and QFC.gb-6B.1 (*R*^2^ = 18.1%).

### QTL Mapping Analysis for Kernel Weight Related Traits and Heading Date

#### Thousand Kernel Weight

Four QTLs associated with TKW were detected on chromosomes 1B, 2B, 3B and 5B explaining 10–22.5% of PEV (Table 4). Notably, the QTL detected on 5B (QTKW.gb-5B) was stable across three environments, explaining 12.3–22.4% of the phenotypic variation. The allele of Iran_249 positively contributed to most of the QTLs.

#### Test Weight

Only two significant QTLs were found both on chromosome 6A and explaining around 18% of phenotypic variance with positive allele contributed by both parents.

#### Heading Date

Seven QTLs for heading date (HD) were detected on chromosomes 2A, 2B, 3B, 4B, 5A, 5B, 7A, and three of these were environmentally stable. In detail, the QTL located on 2B (named as QHD.gb-2B) was conserved among the three sites and explained from 9.9 to 40.6% of variation. Other two QTLs on chromosomes 2A (QHD.gb-2A) and 5B (QHD.gb-5B) were stable in two environments and explained up to 15.6% of PEV. For all these regions, the additive effect responsible for late flowering was contributed by Iran_249, with the only exception of QHD.gb-5A.

### Cluster of QTLs

**Figure 4:**
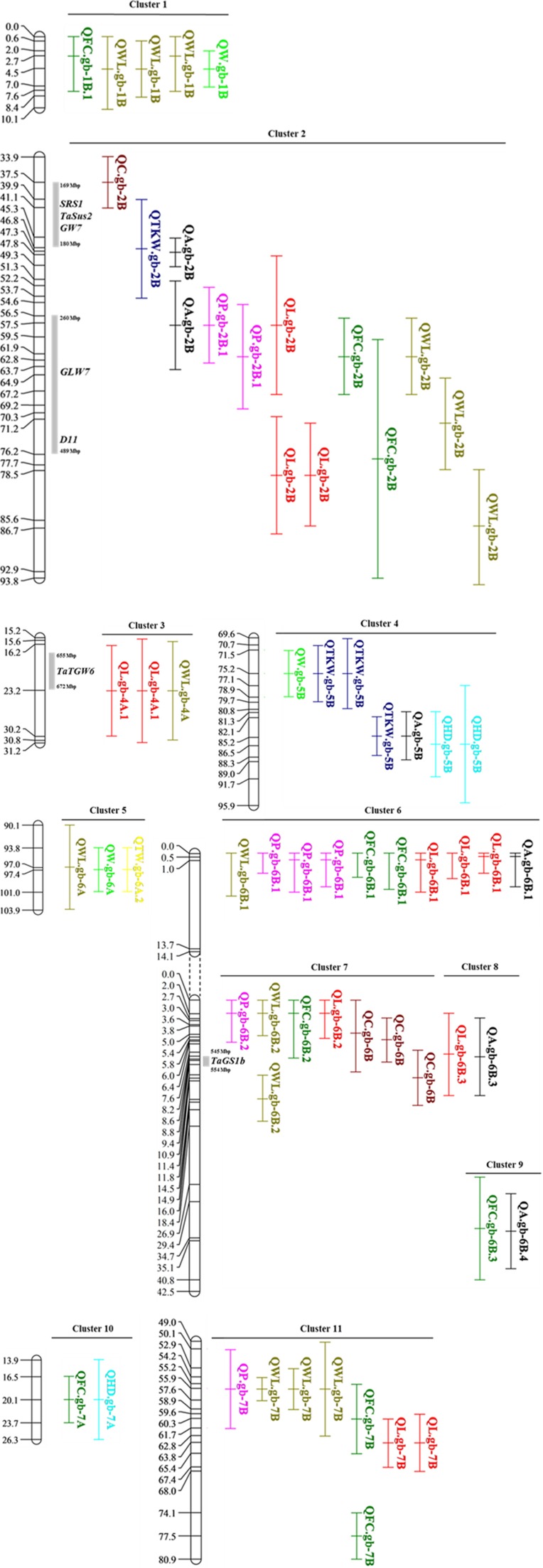
Distribution of co-localized QTLs in clusters detected in this work. Known genes co-localized with our regions have been reported.

Since the parameters used to characterize the kernel are all geometrically or biologically related, we expected to identify coincident loci for different traits. Indeed, the co-localization of QTLs for different traits, proven the coherence about parent providing the QTL additive effect, allowed to define 11 QTL clusters (Table 5 and Figure 4). Clusters included up to 14 QTLs, and the largest clusters were found on chromosomes 2B (cluster 2) and 7B (cluster 11). Regarding cluster 2, the overlapping covered a region that spanned for about 60 cM. We can suppose that this cluster include at least two different associated regions located on chromosome 2B, but based on the resolution of our data they were indistinguishable. Cluster 11 spanned for 30 cM based on two associations for FC whose peaks were located < 20 cM and thus considered the same QTL. Clusters 7 and 8, located on chromosome 6B, were considered as different based on the opposite additive effect values shown by QTL identified for length trait.

**Table 5:** Clusters of QTLs.

**QTL name**	**Environments**	**Peak cM**	***R*^**2**^ (%)**	**CI_start**	**CI_end**	**Cluster**
QFC.gb-1B.1	F15	2.7	14.07	0	7.6	1
QWL.gb-1B	L14, L15, F15	2.7–4.5	12.24–17.7	0	10.1	
QW.gb-1B	L14	4.5	27.66	2	7	
QC.gb-2B.1	L14	37.5	18.97	33.9	41.1	2
QTKW.gb-2B	L15	46.8	10	39.9	53.7	
QA.gb-2B	L15, F15	47.3–57.5	11.1–34.44	45.3	63.7	
QP.gb-2B.1	L14, F15	57.5–61.9	9.49–12.93	52.2	69.2	
QL.gb-2B.1	L15	57.5	7.14	47.8	67.2	
QL.gb-2B.2	L14, F15	78.5	8.47–9.4	70.3	86.7	
QFC.gb-2B	L14, F15	61.9–76.2	4.13–12.7	56.5	92.9	
QWL.gb-2B	L14, L15, F15	61.9–85.6	8.45–12.7	56.5	93.8	
QL.gb-4A.1	L14, F15	23.2	8.68–9.9	15.2	31.2	3
QWL.gb-4A	L14	23.2	9.04	15.6	30.8	
QW.gb-5B	L15	75.2	18.45	71.5	78.9	4
QTKW.gb-5B	L14, L15, F15	75.2–85.2	12.27–22.36	69.6	88.3	
QA.gb-5B	L15	85.2	18.1	81.4	89	
QHD.gb-5B	L14, L15	86.5	7.34–13.3	77.1	95.9	
QWL.gb-6A	F15	97.0	10	90.1	103.9	5
QW.gb-6A	F15	97.4	19.63	93.9	100.9	
QTW.gb-6A.2	F15	97.4	18.97	93.8	101	
QP.gb-6B.2	L14	2.0	16	0.0	6.3	6
QWL.gb-6B.2	L15, F15	2–14.9	19.85	0.0	18.4	
QFC.gb-6B.2	F15	2.0	10	0.0	8.8	
QL.gb-6B.2	L14	2.0	18.18	0.0	5.8	
QC.gb-6B	L14, L15, F15	5.5–11.8	12.78–20.3	0.1	16.0	
QL.gb-6B.3	F15	8.2	10.82	1.9	14.5	7
QA.gb-6B.3	L14	8.6	11.58	2.7	14.5	
QFC.gb-6B.3	L15	34.7	8.83	26.9	42.5	8
QA.gb-6B.4	F15	35.1	11.95	29.4	40.8	
QWL.gb-6B.1	L14	0.0	10.76	0.0	6.4	9
QP.gb-6B.1	L14, L15, F15	0–1.0	14.68–21.51	0.0	5.7	
QFC.gb-6B.1	L14, F15	0.0	12.91–19.29	0.0	5.3	
QL.gb-6B.1	L14, L15, F15	0–1.0	14.77–27.42	0.0	5.6	
QA.gb-6B.1	L15	0.5	15.26	0.0	5.0	
QFC.gb-7A	L14	20.1	19.31	16.5	23.7	10
QHD.gb-7A	L14	20.1	11.1	13.9	26.3	
QP.gb-7B	L14	55.9	11.92	50.1	61.7	11
QWL.gb-7B	L14, L15, F15	55.9	9.95–40.15	49	62.8	
QFC.gb-7B	L15	60.3–77.5	13.52–20.15	55.2	80.9	
QL.gb-7B	L14, F15	63.8	16.41–19.08	59.6	68	

*Each cluster grouped QTLs with overlapping CIs but related to different traits*.

Notably, the overlapping of QTL, also supported by correlation between traits, can suggest causative relationships among the different kernel parameters. Coincidences might derive from different parameters describing the same kernel trait, like WL, FC and C, and thus indicate simple relationships. For instance, the chromosome 6B (clusters 6 and 9) hosted coincident QTLs for all three shape related traits, while three regions on chromosomes 1B, 2B, and 7B were identified using both WL and FC data, and defined clusters 1, 2, and 11, respectively. More intriguingly, QTL co-localizations might depend on geometric relationships between primary characters, like L and W, and the secondary traits like WL, P, A and FC, which directly derive from L and W based on geometric formulas (Table 1). As an example, cluster 1 on chromosome 1B grouped QTL related to traits W, WL and FC, suggesting that phenotypic variation for kernel shape associated to the cluster 1 might depend on width variation. Contrarily, in clusters 2 (chromosome 2B), 3 (4A) and 11 (7B), the kernel length was the primary trait associated to a QTL together with a WL locus, indicating a main effect of kernel length on the grain shape. Finally, other clusters included kernel size/shape QTLs and regions associated to TKW and TW. These co-localizations, together with significant correlation between traits, can suggest causal relationships between the simplest kernel trait, kernel length, width and shape, and the more complex relevant agronomic traits, TKW and TW, which indirectly depend on kernel size. This kind of coincidences was indeed revealed by cluster 2, 4 and 5. In detail, the cluster 2 included QTLs for both TKW and kernel size and morphology (A, P, L, FC and C). Notably, cluster 4 contains the QTKW.gb-5B stable in three environments and QTLs for W and A, but also for HD, highlighting a possible effect of phenology on kernel weight, through an impact on specific kernel dimension. An interesting coincidence was also found in cluster 5 (6A) between TW, WL and W, as expected based on the known positive impact of kernel roundness on TW. Notably, Iran_249 contributes the positive allele at cluster 2 and 4, respectively through an allele with positive effect for kernel length and width, respectively. This finding suggested that increase of kernel size from the landrace might improve important agronomic traits like TKW.

### Analysis of Physical Regions Carrying QTLs Related to Kernel Traits

The recent durum wheat reference genome was used as common framework to compare our results with QTLs related to kernel traits already published. To this aim, the CI of the most consistent QTLs as well as the extreme positions of the QTL clusters were anchored on the Svevo genome assembly through the projection of the associated markers. Analogously, the nucleotide sequences of all known (bread) wheat genes or rice genes related to kernel morphology/weight were used as Blast queries to identify the durum wheat orthologs and define their physical position on the Svevo pseudomolecules (Supplementary Table 6).

The comparison of physical position of QTLs and of these orthologs revealed some interesting overlapping which might suggest worth candidate genes (Figure 4; Table 5 and Supplementary Table 6). When anchored to the Svevo genome sequence, the large cluster 2 on chromosome 2B, including a total of 8 QTLs, encompassed several wheat genes or wheat homologs cloned for their effect on kernel size and weight, namely *TaSus2, SRS1, GW7, GLW7* and *D11*. Interesting coincidences were also found on chromosome 4A and 6B where the *TaTGW6* and *TaGS1a* felt in cluster 3 and 9, respectively. In addition, QL-gb.4A.2 maps at around 3Mb from the candidate gene *6-SFT-A2* (Yue et al., 2015).

To further support these genes as candidates of mapped QTLs, we checked if possible sequence variations at these genes are represented by SNPs of the Illumina 90K wheat SNP BeadChip array which also proved to be polymorphic and mapped within our population. Although most of the candidates were covered by Illumina 90K SNP markers, a polymorphic marker was found only for *TaGS1*, in detail IWB13090 mapped at 8.2 cM on chromosome 6B (linkage group 2) in the Zardak × Iran_249 genetic map. This finding allowed us to genetically map the gene *TaGS1b* under the QTL cluster 9.

To assess the novelty of our results, we compared the clusters identified in this work with known QTLs for related traits reported in tetraploid wheat species. Firstly, we checked the physical position of our clusters on the durum reference genome together with those of the QTL previously reported for kernel shape and size by Russo et al. (2014) and Golan et al. (2015) and recently physically anchored on the durum wheat reference genome by the whole metaQTL analysis conducted by Maccaferri et al. (2019). This analysis did not reveal any overlapping. Analogously, we checked the coincidence of our clusters with the physical positions of QTLs genetically mapped for weight related traits and HD in previous studies in tetraploid wheat and physically defined by Maccaferri et al. (2019) on the Svevo genome. In this case, coincidences were found for all 11 clusters (Table 6). In detail, clusters 2, 4, 5, and 10 included QTLs for TKW, TW and HD located in genome regions where QTLs for the same traits have been already detected. Other clusters (3, 6, 7, 8, 9, 11), which grouped QTLs for kernel morphology and size, co-localized with regions known to be associated with yield related traits, thus remarking the functional/biological relationship between grain size and weight. Finally, for all clusters, except 4 and 9, the correspondence with QTLs for HD was reported suggesting the probable pleiotropic effect of phenology on traits about grain size and weight.

**Table 6:** Co-localization of QTL clusters with known wheat and/or rice genes and known QTLs in tetraploid germplasm related to seed size and shape.

**Cluster**					**Best QTL**		**Known genes**	**Tetraploid QTL/MTA**
**N^**°**^**	**Chr**	**Start (Mbp)**	**End (Mbp)**	**Traits**	**Start (Mbp)**	**End (Mbp)**		
1	1B	8.2	14.6	FC, WL, **W**	6.8	12.5		HD^1^
2	2B	106.7	536.81	C, TKW, **A**, P, L, FC, WL	188.1	222.8	*TaSUS2, SRS1, GW7, GLW7, D11*	TKW^2^, TW^3^, HD^4^
3	4A	628.9	686.7	L, WL	\	\	*TaTGW6*	TW^3^, HD^3^, TKW^5^
4	5B	420.8	489.7	W, **TKW**, A, HD	435.7	452.4		TKW^5^^,^^6^^,^^7^
5	6A	539.4	576.8	**W**, WL, TW	549.5	590.4		TKW^4^^,^^8^^,^ TW^3^^,^ HD ^9^^,^ ^10^
6	6B	2.0	31.3	P, **WL**, FC, L, C	24.4	31.4		TKW^11^^,^^12^^,^ TW^13^^,^ HD^3^^,^ ^9^
7	6B	15.9	26.9	L, **A**	15.9	26.8		TKW^11^^,^ ^12^^,^ TW^13^, HD^14^^,^ ^9^^,^^3^
8	6B	41.8	149.6	FC, **A**	54.9	124.1		TKW^5^^,^ ^6^^,^ ^7^^,^ ^15^^,^ TW^3^^,^^6^^,^ HD^16^^,^^3^^,^^10^
9	6B	552.9	622.6	WL, P, FC, **L**, A	610.7	622.7	*TaGS1b*	TKW^5^^,^^7^^,^^9^^,^^17^
10	7A	673	684.9	**FC**, HD	673.1	683.5		HD^14^
11	7B	606.3	687.9	P, **WL**, FC, L	609.8	633.6		TKW^9^, HD^9^^,^^14^^,^^15^

*The best QTLs are reported in bold*.

1 (Maccaferri et al., 2008);

2 (Faris et al., 2014);

3 (Maccaferri et al., 2011);

4 (Kidane et al., 2017);

5 (Mangini et al., 2018);

6 (Graziani et al., 2014);

7 (Peleg et al., 2011);

8 (Golabadi et al., 2011);

9 (Roncallo et al., 2017);

10 (Milner et al., 2016);

11 (Soriano et al., 2017);

12 (Peng et al., 2003);

13 (Canè et al., 2014);

14 (Maccaferri et al., 2014);

15 (Giraldo et al., 2016);

16 (Elouafi and Nachit, 2004);

17 *(Blanco et al., 2012)*.

For each cluster, the physical region underlined by the CI of the most consistent QTLs was inspected for candidate genes. To this purpose, we took advantage of the durum wheat reference genome (Maccaferri et al., 2019) together with the expression data available for the orthologous bread wheat genes. All predicted genes on the *T. durum* reference genome were functionally annotated through Blast2Go available at FIGSHARE (https://figshare.com/s/2629b4b8166217890971), while for *T. durum* genes lying under the anchored QTLs the *T. aestivum* ortholog was identified. Expression data of these bread wheat genes were retrieved and reported as a heat map in Supplementary Table 7. This approach was expected to support the identification of candidate genes based both on the functional annotation and expression profile in the closely related species *T. aestivum*. Focusing the attention on expression data, grain and spike specific genes have been identified in the genomic regions controlling the following traits: kernel width (chromosome 1B and 6A), kernel length (4B, 6B), kernel area (6B), kernel shape (7B and 7A), and TKW trait (5B). Among these candidate genes, seed and spike specific chromatin remodeling factors (TRITD4Bv1G205360, TRITD5Bv1G146200, TRITD6Av1G202880, TRITD6Bv1G197750, and TRITD7Bv1 G204890), ubiquitin ligases (TRITD5Bv1G144430, TRITD 5Bv1G144440, TRITD6Av1G195410, TRITD6Av1G212580, and TRITD6Av1G212590), and cell wall modeling factors (TRITD6Av1G205500 and TRITD6Av1G205580) might play a role in controlling seed morphology.

## Discussion

Kernel weight and shape are important parameters determining the wheat profitability, being the main determinants of yield and its technological quality. Indeed durum wheat breeding has constantly pursued the improvement of TKW and TW. In parallel, a plethora of studies dissected the genetic bases of TKW and TW in wheat. However, while some works investigated the genetic bases of grain shape and size traits and their relationship with TKW and TW in bread wheat (Sun et al., 2008; Gegas et al., 2010; Ramya et al., 2010; Tsilo et al., 2010; Prashant et al., 2012; Maphosa et al., 2014; Rasheed et al., 2014; Williams and Sorrells, 2014; Wu et al., 2015; Kumar et al., 2016; Cheng et al., 2017; Würschum et al., 2018), very few were dedicated to durum wheat (Russo et al., 2014; Golan et al., 2015). Moreover, only a few studies based on the linkage mapping approach (Russo et al., 2014; Wu et al., 2015; Kumar et al., 2016; Cheng et al., 2017; Su et al., 2018) used a high-density genetic map to analyze kernel size related traits. Therefore, an understanding of the genetic basis of kernel size/shape traits is an important objective whose results could be deployed in future (durum) wheat breeding. Furthermore, this study was also conceived to inspect the relevant genetic diversity present in less cultivated materials, such as landraces. Therefore, a RIL population derived from a cross among two Iranian durum wheat genotypes, a landrace and a local old cultivar (Iran_249 and Zardak, respectively), was used to investigate durum wheat kernel morphology factors and their relationships with kernel weight, and to map the corresponding QTLs. The two genotypes derive from different regions of Iran and show significant differences for morphology of kernel and spike, with Iran_249 being similar to *T. turanicum*. This wheat, currently cultivated in Iran, is a tetraploid subspecies also called Khorasan wheat, but it is genetically not dissimilar from durum landrace as shown in Maccaferri et al. (2019). For our analysis, we considered the most common parameters used to describe kernel size and shape, in the above mentioned genetic studies, and we applied high-throughput phenotyping based on digital image analysis to get accurate scoring data from a higher amount of seeds per samples from two experimental sites, thus addressing the variability present in seed sample as well as the environment effect. In addition, a high density genetic map, comprising 6,195 markers, was developed and used to perform the QTL analysis. Lastly, we anchored the mapped QTLs on the recently released *T. durum* reference genome.

The experimental field provided phenotypic data that highlight significant variability for the genotype effect for all traits considered, thus allowing to conduct QTL analysis on each single environment data. As possible for large field trial that may likely encompass non-uniform soil parameters, the replicate effect was also significant for almost all traits, however most of the variation was accounted by the genotype component.

About overall data across the three environments, all effects (G, E, and GxE interaction) were significant for all traits, with E accounting for most of the variability. The two sites used for field trail represent two durum wheat growing areas in Italy characterized by strong differences in soil fertility and climatic conditions (Supplementary Table 1). Consequently, for some traits known to be influenced by environment (like HD, TW and TKW), a large environmental effect, even larger than the genotype component, was observed, that is large differences among environmental means causing most of the variation in genotype performances. This confirms that the experimental sites were enough different to highlight the environment and possible GxE effect on the target traits. We can suppose that major differences in these trait phenotypes were associated to rainfall levels and temperature values. This was already reported in studies about the performances of durum genotypes conducted in the same two experimental sites (De Vita et al., 2010), and in general for the target traits across different environments (Graziani et al., 2014; Kumar et al., 2016). The observation that environment affects also kernel width, and consequently WL ratio and FC, may suggest that the environment impacts on TKW and TW through effects on width of kernel, and in a minor extent through length of kernel. More interesting is the impact of GxE interaction on total variation. We found significant GxE variability for almost all traits, but interactions contributed significantly less to the phenotypic variations, compared with the genotypic effects. Indeed, we were able to identify QTLs stable across the three environments. The only exception is TW that, with a GxE variance higher than that due to genotype, revealed its low heritability level. Accordingly, lowest level of heritability was observed for kernel width which is the morphology trait more correlated with TW. Previous studies have already reported lower level of heritability for width of kernel in comparison to length of kernel (Russo et al., 2014; Kumar et al., 2016; Su et al., 2018), thus length promises to be an effective target for breeding.

Correlations among size and shape related traits, as well as with kernel weight have been addressed in all the mentioned studies in wheat, aiming to highlight distinct genetic controls and to disentangle complex traits in their simplest but likely causative primary traits. In our case, we observed positive correlation between size related traits and grain weight, a higher correlation of width with weight of kernels as opposed to length of kernels, and a negative correlation among kernel width and kernel length. These observations, in agreement with insights from previous studies (Russo et al., 2014; Kumar et al., 2016; Cheng et al., 2017; Su et al., 2018), suggest that kernel width should be the main contributor to the increased grain weight and that kernel length and width are probably under different genetic control. Analogous results have been obtained in bread wheat, through a detailed analysis which has dissected the phenotypic and genetic structure of kernel size and shape (Gegas et al., 2010). The authors developed a phenotypic model integrating grain size and shape parameters, thus demonstrating that the kernel length and width traits are probably under the control of distinct genetic components.

The present study identified a total of 94 QTLs along all chromosomes; in detail, 43 QTLs for traits related to kernel size (L, W, P, A), 32 QTLs associated with kernel shape (C, WL, and FC), 8 QTLs for kernel weight (TKW and TW) and 11 regions associated with heading date. The phenotypic variation explained by each QTL ranged from 4.1% (QFC.gb-2B) to 40.1% (QWL.gb-7B), with an average of 15.7%. Thus, both few major and several minor QTLs for all the grain characteristics were identified, confirming the polygenic control of these traits suggested by the distribution of phenotypic data as already reported. Many of the QTLs identified were environment specific as expected according to the significant GxE effect observed for all traits. However, we were able to identify robust QTLs stable across two or three environments. Indeed, three regions associated to WL (QWL.gb-1B, QWL.gb-2B, and QWL.gb-7B), two regions identified by length data (QL.gb-2B and QL.gb-6B.1) and one region for P (QP.gb-6B.1), C (QC.gb-6B), HD (QHD.gb-2B) and TKW (QTKW.gb-5B) were effective in all evaluation trials. Other 14 QTLs detected for traits A, C, P, FC, HD, L, and WL, and spanning on different chromosomes (2A, 2B, 4A, 4B, 5B, 6B, and 7B) were expressed in two environments.

Focusing on the parent contribution, Iran_249 contributed the allele with increasing effect for most of the QTLs related to kernel length, vice versa for QTLs related to kernel width is Zardak the parent conferring the allele with positive effect. Moreover, Iran_249 conferred positive allele at 4 out of 6 loci related to kernel weight (TKW and TW), although kernel width showed consistently higher positive correlation with kernel weight than kernel length. Landraces are considered valuable resource to enlarge the genetic diversity of modern cultivated genetic pools (Moore, 2015), however, to the better of our knowledge, the variance available for kernel length has been rarely addressed so far for the landraces, neither in durum wheat nor in bread wheat (Abdipour et al., 2016). For common wheat, a detailed analysis of the kernel size and shape trait assessed the genetic variation available among/within wheat subspecies, including primitives. In contrast to modern wheat varieties, these primitives exhibited broader variation in grain size and shape with grain width being the least variable trait, meaning that the modern breeding germplasm has lost grain morphology variation, probably due to selection for more uniform grain shape in the élite varieties (Gegas et al., 2010). In this context, our finding suggest that landraces, as exemplified by Iran_249, might have considerable potential toward enhancing the existing gene pool for grain shape and size traits and for further yield improvement in wheat, without the issue of linkage drag related to using primitive wheat's.

QTLs identified in the present study were grouped according to their genetic positions and the parent responsible of positive additive effect, thus identifying 11 cluster regions which include both loci for the primary traits L and W, as well as their corresponding derivative traits (WL, P, A and FC), and relevant agronomic traits (TKW, TW and HD). QTL clustering or coincidence is common in wheat for a number of traits and has been already reported for kernel morphology and weight (Gegas et al., 2010; Russo et al., 2014; Wu et al., 2015). It suggests that associated loci either have pleiotropic effect or are closely linked, both resulting in phenotypic correlations among corresponding traits (Kumar et al., 2016; Cheng et al., 2017). The cloning of several genes for grain shape and size genes in rice and wheat also confirmed the pleiotropic effects of those genes (Fan et al., 2006; Song et al., 2007). Following this rationale, because of the geometric and/or physiologic relationship among the traits, clusters are expected to suggest which primary kernel trait, between kernel length and width, might more strongly impact on a co-located and more complex secondary trait, like kernel shape and, more intriguingly, weight related traits. For instance, based on this assumption, we could hypothesize that for the cluster 1 (chromosome 1B) phenotypic variation for WL might depend on the co-located QTL for kernel width. Analogously, in clusters 2 (2B), 3 (4A), and 11 (7B) variation for length was likely responsible for the identification of the WL and FC loci. Notably, the comparison of the regions associated to TKW with QTLs for kernel size might identify relevant relationships between kernel size and yield related traits. This coincidence was revealed by two clusters, evidencing effect of both kernel length (cluster 2 on 2B) and width (cluster 4 on 5B) on kernel weight. Interestingly, cluster 4 contains the robust QTKW.gb-5B, repeatedly identified in three environments, QTLs for W and A, but also for HD, highlighting a possible effect of phenology on kernel weight, putatively through an impact of regulation of HD on specific kernel dimension (W). Another interesting coincidence might indicate the positive impact of kernel roundness on TW. This is the case of cluster 5 (6A) which included QTLs for TW, WL and W. Notably, Iran_249 contributes the positive allele at cluster 2 and 4, respectively through an allele with positive effect for kernel length and width, respectively. As we already pointed out, this finding suggested that increase of kernel size from the landrace might improve important agronomic traits like TKW.

The projection of the clusters on the *T. durum* reference genome sequence allowed to enlarge this approach considering QTLs for the target traits so far mapped in tetraploid wheat germplasm. Within all clusters some potentially coincidences emerged with QTL previously identified through both linkage and association mapping and recently physically mapped on the reference genome (Maccaferri et al., 2019). Interestingly, most of our clusters of QTLs for kernel morphology and size co-located with known QTLs for TKW and TW (Peng et al., 2003; Elouafi and Nachit, 2004; Maccaferri et al., 2008; Peleg et al., 2011; Canè et al., 2014; Graziani et al., 2014; Roncallo et al., 2017; Soriano et al., 2017; Mangini et al., 2018). This result further indicates that kernel size/shape genetic determinants are responsible for variability in kernel weight, suggesting that selection for these traits can indirectly improve grain weight. In other cases, coincidence was found between QTLs for the same traits, thus validating the results shown in the present study, also for those QTLs that are expressed only in one environment. For example, Faris et al. (2014) and Mangini et al. (2018) reported QTL for TKW on chromosome 2B that may correspond to QTKW.gb-2B, while QTKW.gb-5B and QTW.gb-6A could correspond to the QTLs previously reported on chromosomes 5B and 6A for the same traits (Maccaferri et al., 2011; Peleg et al., 2011; Graziani et al., 2014; Mangini et al., 2018).

The recent release of the *T. durum* reference genome (cv. Svevo) allowed us to identify durum wheat homologs to rice genes known to be involved in the regulation of kernel size and weight [as summarized by Huang et al. (2013); Li and Yang (2017)] as well as new candidate genes. The base assumption supporting this approach is that most of the gene content is conserved among the cv. Svevo and parental lines selected for this study. Consequently, the diversity observed in the current work is supposed to be mainly related to allelic variation at conserved loci, at to a lesser extent to different gene content. The large cluster 2 on chromosome 2B, including 8 QTLs detected for kernel size/shape (C, A, P, L, FC) and weight traits (TKW), encompassed several wheat genes or wheat homologs cloned for they effect on kernel size and weight (*TaSus2, SRS1, GW7, GLW7* and *D11*). In details, *TaSus2*, involved in the starch synthesis pathway had a direct association with grain yield in wheat representing one of the major target of indirect selection in wheat breeding for higher yield. Other cloned rice genes (*SRS1, GW7, GLW7* and *D11*) appear to be involved directly in the seed morphology, by determining the spatial control of cell division, and indirectly in the regulation of yield. However, the extension of cluster 2 impaired us to hypothesized which gene represents the best candidate gene involved in the trait determination. Interesting coincidences were also found on chromosome 4A and 6B where the *TaTGW6* and *TaGS1b* felt in cluster 3 and 9, respectively. We were also able to genetically map the *TaGS1b* gene in the Zardak × Iran_249 population under the QTL cluster 9. Both these genes were associated to high grain weight but for the homeolog form *TaGS1a* functions for grain size and shape functions have been also hypothesized (Bernard et al., 2008; Guo et al., 2013). Therefore, the QTL present in cluster 9 as associated to kernel size could correspond to *TaGS1b*.

Besides known genes, novel candidates emerged by inspecting the gene content of the genomic regions underlined by the most consistent QTL for each cluster. Both functional annotation and expression data, as predicted based on the tissue specific RNA-seq data available for *T. aestivum*, were considered. Among the tens of genes located under the target QTLs, we were able to find a subset of genes specifically expressed in grain and spikes and having functional annotation already reported for genes related to grain size and shape in rice and wheat. However, fine mapping approaches together with detailed expression profile analysis in the parental lines are required to increase the mapping resolution and thus identify best candidate genes.

In the most recent breeding above all, the market and industry requirements for almost spherical grains led to selection for larger grains. However, yield was unaffected due to reduced kernel number (Wiersma et al., 2001), as consequence of the physiological trade-offs between individual components of yield (kernel number, kernel weight, kernel shape, etc.). These complex physiological relationships hinder improvement of grain yield when trying to manipulate single yield component using only phenotypic data. The knowledge of the genetic bases of such complex quantitative traits, together with relevant new alleles from less cultivated germplasm can contribute to model the interactions among components, to find effective combinations of traits and candidate genes, toward the improvement of wheat kernel size and yield.

## Author Contributions

FD and EM carried out the data analyses and wrote the manuscript. FD, EM, DG, SL, NV, and FS participated in field and post-harvest evaluations of phenotypic traits. PB and RB performed the bioinformatic analysis. LZ, KC, and EF developed RIL population. FD, MP, LC, and EM designed the study. All authors have read and approved the final manuscript.

### Conflict of Interest Statement

The authors declare that the research was conducted in the absence of any commercial or financial relationships that could be construed as a potential conflict of interest.

## Footnotes

^1^Winseedle pro (2011 Regents Instruments Inc., Canada).

## Supplementary Material

Supplementary Figure 1 Pearson correlations among the phenotypic traits analyzed using single environment data: L14 **(A)**, L15 **(B)**, and F15 **(C)**.

Supplementary Table 1 Field experimental conditions and environmental parameters.

Supplementary Table 2 Summary of phenotypic data and variation parameters for parental lines and RILs for kernel shape, size and weight related traits, and heading date. Mean data for each environment (L14, L15, and F15) has been reported.

Supplementary Table 3 Variance components of RIL population for each trait using both mean data across the three environments and data from single environments (L14, L15, and F15).

Supplementary Table 4 Pearson correlations and corresponding p-value, among the phenotypic traits analyzed using both overall data and data from single environments (L14, L15, and F15).

Supplementary Table 5 Zardak × Iran_249 linkage map. Linkage groups, chromosomes, marker names and their position (cM, obtained using the Kosambi mapping function) are reported.

Supplementary Table 6 Genes controlling kernel related phenotypes (TKW, L, W) identified in wheat and/or rice. Gene name, gene ID, wheat chromosome and the physical position on the *T. durum* reference genome are reported.

Supplementary Table 7 Gene content under the selected QTL regions. Functional annotation and expression data are also reported. Expression data related to the ortholog bread wheat genes were downloaded from the WheatExp database (https://wheat.pw.usda.gov/WheatExp/). Tissues and stages are the following: Grains collected at the Zadoks scale 71, 75, and 85, whole endosperm collected at 10 and 20 days After Pollination (DAP), endosperm tissues as starchy endosperm, transfer cells, the aleurone, maternal tissues as the inner and outer pericarp, spikes collected at the Zadoks scale 32, 39, and 65, leaves collected at the Zadoks scale 10, 23, and 71, root collected at the Zadoks scale 10, 13, and 39, and stem collect at the Zadoks scale 30, 32, 65.
