# Supplementary figures and images for: Genomic Regions From an Iranian Landrace Increase Kernel Size in Durum Wheat
Source: Front Plant Sci. 2019 Apr 18;10:448. doi: 10.3389/fpls.2019.00448 (PMC6482228; doi:10.3389/fpls.2019.00448)

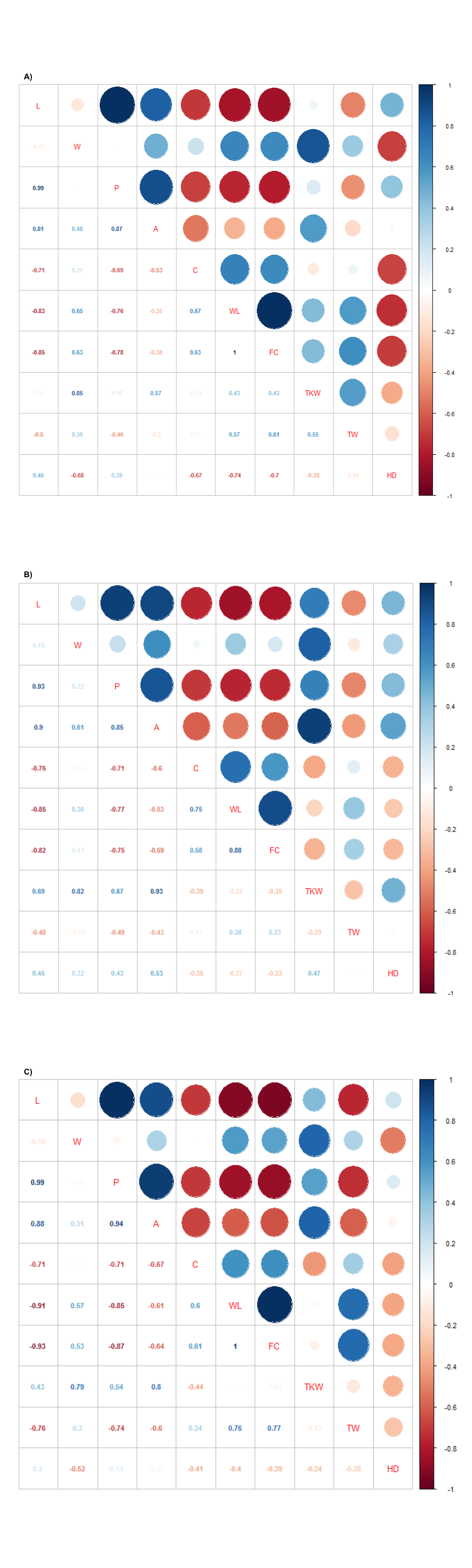

Supplement: Supplementary Figure 1 — Pearson correlations among the phenotypic traits analyzed using single environment data: L14 (A), L15 (B), and F15 (C). [file Image_1.TIFF]
